# Native nephrectomies in patients with autosomal dominant polycystic kidney disease: retrospective cohort study

**DOI:** 10.1007/s11845-024-03778-3

**Published:** 2024-08-12

**Authors:** Richard Edmund Hogan, Barry McHale, Gavin Paul Dowling, Elhussein Elhassan, Conor James Kilkenny, Ponnusamy Mohan, Peter Conlon

**Affiliations:** 1 Department of Nephrology and Transplant, Beaumont Hospital, Beaumont Rd, Dublin, Ireland; 2 Department of Medicine, Royal College of Surgeons in Ireland, 123 St Stephen’s Green, Dublin 2, Ireland

**Keywords:** ADPKD, Nephrectomy, Outcomes, Transfusion, Transplant

## Abstract

**Background:**

Approximately 1 in 5 patients with autosomal dominant polycystic kidney disease (ADPKD) will undergo a native nephrectomy in their lifetime. These can be emergent or planned and the indications can range from space for kidney transplant, pain, hematuria and frequent urinary tract infections (UTIs). Due to the diverse nature of presentations, there is a lack of certainty about outcomes and optimal management.

**Aims:**

This study aimed to evaluate preoperative indications and perioperative/postoperative complications in this patient cohort.

**Methods:**

This retrospective review included 41 patients with ADPKD who underwent unilateral or bilateral nephrectomy in a single hospital between 2010 and 2020. We collected data on patient demographics, surgical indications, histological results and postoperative complications. We sourced this information using the hospital’s patient medical records.

**Results:**

The main indications for nephrectomy were pain (39.5%) and bleeding (41.8%). Further indications included recurrent UTIs (16.3%), space for transplantation (27.9%), query malignancy (4.7%) and compressive gastropathy (2.3%). With regard to side, 55.8% were right-sided, 23.3% were left-sided, and 20.9% were bilateral. Seven percent of nephrectomy specimens demonstrated malignancy. Postoperative morbidity included requiring blood transfusion and long hospital stay. Thirty-seven percent of patients received a postoperative blood transfusion. There was no immediate or postoperative mortality associated with any of the cases reviewed.

**Conclusions:**

In conclusion, this study demonstrates that native nephrectomy remains a safe operation for patients with ADPKD. Although further research is needed into, transfusion protocols, adjunctive therapies, such as TAE and research into timing of nephrectomy are still needed.

## Introduction

**Fig. 1:**
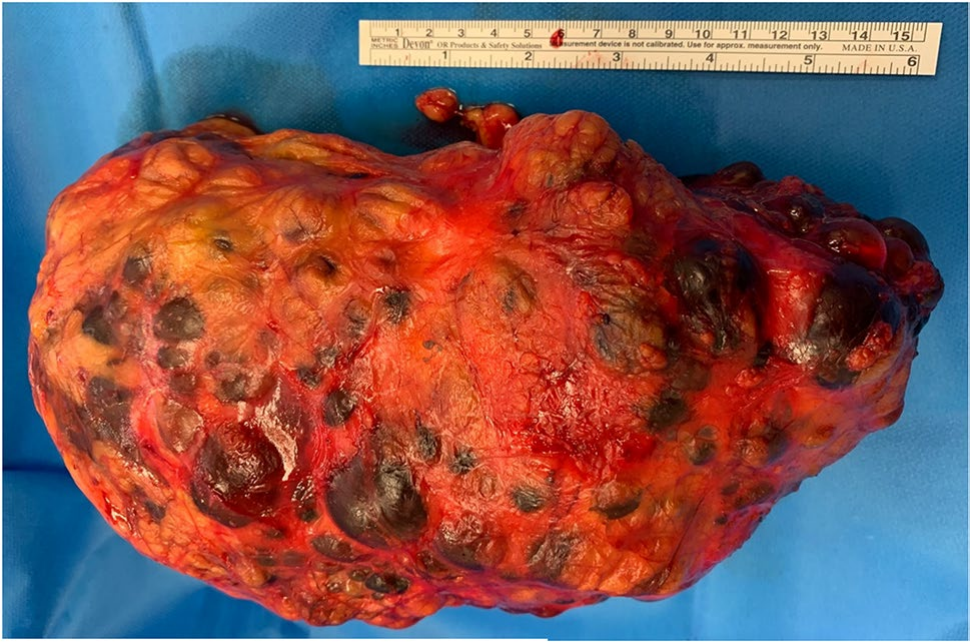
ADPKD specimen

Autosomal dominant polycystic kidney disease (ADPKD) is the most common genetic cause of end stage renal disease globally [1, 2]. It is characterized by the presence of numerous fluid-filled cysts in both kidneys (Fig. 1). Cyst growth can complicate disease course by causing pain due to cyst rupture, haemorrhage, infection, stone or urinary tract infection (UTI) [1]. In symptomatic patients with ADPKD, nephrectomy might be indicated if symptoms persist despite appropriate medical treatment. Approximately one in five patients with ADPKD will receive a native nephrectomy in their lifetime [3]. The main indications for native nephrectomy in patients with ADPKD are abdominal pain, haematuria and anaemia, space for transplant, chronic UTI and suspected renal malignancy [3].

Native nephrectomy can be performed either laparoscopically or via an open approach. In the past 20 years, there has been a large increase in the amount of laparoscopic native nephrectomies performed for ADPKD [4–6]. However, some centres still primarily use the open approach, such as in the centre where this study was performed.

Removing large polycystic kidneys might pose more surgical risks, such as increased blood loss, prolonged operative time and higher conversion into an open approach. When removing large polycystic kidneys, a surgeon may prefer the open approach to visualise and remove the kidney. A systematic review by Guo et al., comparing the open vs laparoscopic approach in patients with ADPKD, showed that even in large symptomatic kidneys the laparoscopic approach was safe and feasible [4].

Traditionally, native nephrectomy prior to transplantation was the accepted standard. In recent years, simultaneous nephrectomy and transplantation have grown in popularity in some centres around the world, while others have continued to perform two separate surgeries [7–11]. A recent systematic review in 2021 analysed whether timing nephrectomies, staged versus simultaneous, with transplantation in patients with ADPKD, would affect operative or graft outcomes [8]. It was unable to find a statistically significant difference between graft thrombosis in either group [8]. Thus timing and indication for nephrectomy remain open to debate.

In this retrospective study, we analysed the outcomes from native nephrectomies without simultaneous transplantation in patients with ADPKD. We focused on reviewing indication of surgery, peri- and postoperative outcomes, and number of patients undergoing kidney transplant after polycystic kidney nephrectomy.

## Material and methods

The Beaumont Hospital clinical research and audit department approved this retrospective review (CA2022/074). Data from all patients, with ADPKD, who underwent a native nephrectomy between January 2010 and December 2020 were collected.

The inclusion criteria for this study were limited to adult ADPKD patients who underwent native nephrectomy. We excluded those with partial nephrectomy, transplant nephrectomy, and non-ADPKD genetic kidney disease.

### Data collection

Detailed data regarding patient demographics, preoperative stage of chronic kidney disease (CKD), dialysis status, surgical indication, histological information, surgical procedure, time to transplant afterwards and postoperative complications were collected using patient medical records. Furthermore, we gathered data on the number of units of RCC transfused and pre- and postoperative haemoglobin. We sourced this information using the hospital’s electronic records of laboratory results, imaging and histology reports and patient medical records.

### Statistical analysis

Categorical data are presented as counts and percentages with continuous data presented as mean and standard deviations. Descriptive statistical analysis was performed using SPSS version 22 (IBM SPSS, Armonk, NY, USA).

## Results

###    Patient demographics

During the study period, 43 native nephrectomies were performed, with 2 patients receiving two native nephrectomies in two separate operations. A total of 41 patients with ADPKD were included. Forty-one of the forty-three nephrectomies were performed with the open approach with two performed laparoscopically. This was due to surgeon preference in the study centre. There were 17 (39.5%) male patients and 26 (60.5%) female patients. The mean age at time of surgery was 53.4 ± 10.1 years. Of the 43 operations performed, there were 24 right-sided nephrectomies, 10 left-sided and 9 were bilateral. The average weight of a kidney removed at histology was 1855 ± 1203 g and the largest kidney removed weighed 6014 g. These demographics are summarised in Table 1.

**Table 1:** Demographics

*N*	43
Age (years)	53.4 ± 10.1
Gender (M/F)	17/26
Kidney	
Right	24 (55.8%)
Left	10 (23.3%)
Bilateral	9 (20.9%)
Weight (gr)	1855.5 ± 1203

### Indication for surgery

The most common indications for surgery were chronic bleeding (18/43; 41.8%) and pain (17/43; 39.5%). The indications for surgery were not mutually exclusive with many operations being performed with more than one indication. Space for transplantation was indicated in 12 (27.9%) of the nephrectomies performed. More detail is listed in Table 2.

**Table 2:** Indication

*N*	43
Bleed	18 (41.8%)
Pain	17 (39.5%)
UTI	7 (16.3%)
Space	12 (27.9%)
Query malignancy	2 (4.7%)
Obstructive gastropathy	1 (2.3%)

### Postoperative data and complications

Overall, there was no mortality intraoperatively or during the postoperative stay for all 43 nephrectomies. The average postoperative stay was 10.1 ± 4.5 days. Preoperative and postoperative haemoglobin (Hb) levels were recorded. The average preoperative Hb was 11.1 ± 1.7 g/dl. The average post op Hb was 9.4 ± 1.7 g/dl. The average drop in Hb 1 day after surgery was 1.7 ± 1.5 g/dl. Four patients received a transfusion preoperatively, with an average of 4.25 ± 2.1 units of red cell concentrate (RCC) given per patient. Sixteen patients received a transfusion postoperatively, with an average of 3.7 ± 3.4 units of RCC given per patient.

Of the 41 patients, 6 had received a renal transplant prior to native nephrectomy. At the time of reporting, 19 patients had received a renal transplant since their native nephrectomy and 16 had not yet received one. On histology, 3 (7.0%) nephrectomy specimens were positive for papillary renal cell carcinoma. A summary is shown in Table 3.

**Table 3:** Transfusions

*N*	43
Preoperative	
Average Hb (g/dl)	11.1 ± 1.7
Number of patients transfused	4 (9.3%)
Average units of RCC transfused	4.25 ± 2.1
Postoperative	
Average Hb day 1 (g/dl)	9.4 ± 1.7
Number of patients transfused	16 (37.2%)
Average Hb drop day 1 (g/dl)	1.7 ± 1.5
Average units of RCC transfused	3.7 ± 3.4

*N* number, *M/F* male/female, *gr* gram. *N* number. *N* number, *RCC* red cell concentrate, *Hb* Haemoglobin, *g/dl *grams per decilitre

## Discussion

The most important findings from this study were that patients with ADPKD who undergo native nephrectomy have good overall outcomes and it remains a safe surgical option. However, these patients had long hospital stays and a high rate of blood transfusion. The indications for surgery gathered from our study are consistent with the literature on native nephrectomies in patients with ADPKD [3, 12]. Of note, bleeding was the most common indication for surgery in our study, reported in 41.8% of patients. Transcatheter arterial embolization of the renal arteries (TAE) is a non-invasive procedure utilised in patients with symptomatic polycystic kidneys to help reduce kidney volume, pain and reduce bleeding [5, 13, 14]. It has been shown to be safe and effective in order to optimise patients for kidney transplantation [14]. Given the high rate of reported bleeding in our study, it shows that further research into TAE as an alternative approach to manage these patients is needed. A study by Akabane et al. investigates using TAE combined with nephrectomy [13]. This would have the added benefit of decreasing kidney size before nephrectomy allowing for easier access and potentially lower complications.

Although research into laparoscopic nephrectomies has shown favourable outcomes, even for massive kidneys, the majority of surgeries within our institution were performed using an open approach. The recommendation from the literature is for optimal choice to be made from the surgeon’s experience [5]. Our study reported no deaths in the immediate postoperative period. This is important to highlight as it demonstrates that open nephrectomy is still a safe procedure if required for patients. The centre where this study was performed is also the national centre for kidney transplantation. The surgeons operating in this centre are familiar with the open approach for transplant and nephrectomy. Although recent evidence does suggest that the laparoscopic approach may have more favourable outcomes, what is most important is surgeon experience; this is likely why majority of nephrectomies in ADPKD patients in our hospital were performed using the open approach. Our study shows that the open approach is still a safe operation to offer patients. The open approach has also been shown to have shorter operation times [15]; it is likely in some busy centres, where multiple specialties are competing for theatre space, that this may influence the surgeon’s choice of approach.

The average length of stay (LOS) in this study was 10.1 days. This is a comparable figure to other studies on open nephrectomy in ADPKD patients, with reports ranging from 6.5 to 12 days [3, 7, 16, 17]. The reported LOS after laparoscopic nephrectomy is significantly shorter in the literature, reported at 4–8 days [4, 16–18]. In a study published by Lubennikov et al., comparing the open with the laparoscopic approach for nephrectomies in ADPKD, they found a median length of stay for the open approach of 12.5 days [17]. They further broke their results into emergent and elective operations and found an elective LOS of 9 days and 16.5 for emergent operations. In our study, we did not further subdivide our groups into elective and emergent due to the risk of adding bias. Majority of these operations are performed with a certain level of urgency, especially with haemorrhage being the largest indication, we felt that separating these patients into groups retrospectively would add observer bias as to what was elective and what was an emergency and thus may create unreliable results. This likely explains are mean LOS of 10.1 days being on the higher range of that reported in the literature.

Our results found a malignancy rate of 7% among histology specimens. A recent systematic review by Drake et al. found a malignancy rate of 5.7% among patients with ADPKD [19]. Our results are consistent with the current literature regarding RCC in patients with ADPKD.

Blood transfusion in patients with ADPKD has been a widely debated topic for a number of years [20]. The literature would recommend minimising all transfusions in this population. This is due to HLA sensitization and the resultant effect it may have on future transplantation [20]. Current evidence would suggest that multiparous women, patients with previous allografts and patients who have received multiple blood transfusions in the past are most at risk of HLA sensitization after receiving a blood transfusion [20]. Administration of EPO and other blood-conserving treatments is essential to minimise transfusion. There is no specific guideline on transfusion thresholds in these patients postoperatively. Instead, a patient-specific approach is recommended by analysing all the above risks, along with other co-morbidities such as age, previous strokes or coronary artery disease [20]. Blood transfusions are classified as a grade 2 complication with the Clavien-Dindo Classification for postoperative complications [21]. This study reported 16 (37.2%) patients receiving a transfusion postoperatively. This is comparable to reports from other studies, ranging from 0 to 42.4% [10, 12, 22]. A study by Eng et al. found an overall postoperative transfusion rate for open nephrectomy in ADPKD patients of 41.7%, and another study by Verhoest et al. found a postoperative transfusion rate of 15.8% [23, 24]. Our study reports a rate of 37.2% which is consistent although on the upper range of what other studies have reported. It is clear that the rate of transfusion ranges quite significantly from one study to another, and this is likely due to the lack of consensus among transfusion targets in this population. It is clear that further research and consensus on blood transfusion in this population is necessary in the future to formulate a clear perioperative transfusion guideline for these patients.

In this retrospective study, 6 patients had already received a renal transplant prior to nephrectomy. A further 19 (46.3%) patients went on to receive a renal transplant postoperatively. This study did not follow up the outcomes of these patient’s post-transplant and thus information on timing of nephrectomy in relation to transplant cannot be gained from this study. Current research into simultaneous transplantation with nephrectomy has shown comparable outcomes while reducing the number of operations performed on patients [8].

## Limitations

This study is not without its limitations. This study is retrospective in nature and therefore all limitations inherent to such study design apply to the current study. Additionally, our study was unable to gather more detailed data on postoperative complications included in the Clavien-Dindo Classification. Finally, this study represents patients from a single-centre cohort, which has the potential to limit generalizability.

## Conclusion

In conclusion, this study demonstrates that native nephrectomy remains a safe operation for patients with ADPKD, although further research into transfusion protocols, adjunctive therapies, such as TAE, and research into timing of nephrectomy are still needed.

## Data Availability

This was a retrospective chart review of already available data.
